# Phylogenetic Relatedness within the Internally Brooding Sea Anemones from the Arctic-Boreal Region

**DOI:** 10.3390/biology10020081

**Published:** 2021-01-22

**Authors:** Anita Kaliszewicz, Ninel Panteleeva, Magdalena Żmuda-Baranowska, Karol Szawaryn, Izabella Olejniczak, Paweł Boniecki, Sergey D. Grebelnyi, Dagmara Kabzińska, Jerzy Romanowski, Rafał Maciaszek, Ewa B. Górska, Joanna Zawadzka-Sieradzka

**Affiliations:** 1 Institute of Biological Sciences, Cardinal Stefan Wyszyński University in Warsaw, 01-938 Warsaw, Poland; iza-olejniczak@wp.pl (I.O.); pawbon@wp.pl (P.B.); j.romanowski@uksw.edu.pl (J.R.); asiaz76@o2.pl (J.Z.-S.); 2 Murmansk Marine Biological Institute, Russian Academy of Sciences, 183010 Murmansk, Russia; ninel_panteleeva@mail.ru; 3 Institute of Physical Chemistry, Polish Academy of Sciences, 01-224 Warsaw, Poland; mzb.eduscience@gmail.com; 4 Museum and Institute of Zoology, Polish Academy of Sciences, 00-679 Warsaw, Poland; el.cahir@gmail.com; 5 Zoological Institute, Russian Academy of Sciences, 199034 Saint Petersburg, Russia; sgrebelnyi@gmail.com; 6 Neuromuscular Unit, Mossakowski Medical Research Centre, Polish Academy of Sciences, 02-106 Warsaw, Poland; dagkab@imdik.pan.pl; 7 Department of Animal Genetics and Conservation, Institute of Animal Sciences, Warsaw University of Life Sciences, ul. Ciszewskiego 8, 02-786 Warsaw, Poland; rafal_maciaszek@sggw.edu.pl; 8 Department of Biochemistry and Microbiology, Institute of Biology, Warsaw University of Life Sciences SGGW, 02-787 Warsaw, Poland; ewa_gorska@sggw.edu.pl

**Keywords:** internal brooding, *Urticina*, *Cribrinopsis*, Arctic-boreal species, phylogenetic relationships, phylogeny

## Abstract

**Simple Summary:**

Sea anemones owe their phylogenetic uncertainty to the lack of correspondence between taxonomy and morphological and biological traits. We focused on the phylogenetic relationships within the genera *Urticina*, *Cribrinopsis*, and *Aulactinia* including brooding Arctic-boreal species that are found in aggregations in intertidal and subtidal zones. Nuclear 28S ribosomal DNA partial sequences were desirable for analyses of taxonomic relationships between these genera. Mitochondrial and morphological genealogies did not appear to be representative and sufficient for separating taxa lower than the level of families. Despite brooding strategy has been described as increasing offspring survival but decreasing dispersal potential, the dispersion of the juveniles of the studied Arctic-boreal species might be sufficient to settle remote habitats.

**Abstract:**

Phylogenetic analyses based on mitochondrial 16S rDNA, nuclear 28S rDNA, and morphological and ecological traits of *Aulactinia, Urticina* and *Cribrinopsis* sea anemones inhabiting the Arctic-boreal region indicate discordances between trees derived from molecular sequences and those based on morphological traits. Nuclear genes were more informative than mitochondrial and morphological datasets. Our findings indicate that 16S rDNA has limited applicability for phylogenetic analyses at lower taxonomic levels and can only be used for distinction of families. Although 28S rDNA allowed for the classification of distinct genera, it could not confirm that species of *Urticina* and *Cribrinopsis*, which appeared to be closely related, were correctly separated into two different genera. The nuclear tree revealed inconsistencies between specimens belonging to European *Urticina crassicornis* and Pacific *U. crassicornis*; the latter seems to be a different species. In contrast to Pacific *U. crassicornis*, the specimens collected from different localities in the Barents Sea are on the same tree branch. The same was observed for specimens of *Aulactinia stella*. Both species brood their young internally. The dispersal of sea anemones with brooding juveniles seems to be less limited than expected and might be sufficient to settle habitats more than a thousand kilometers away.

## 1. Introduction

There is still a large group of animals that, despite their wide distribution in the world’s oceans, remain taxonomically disordered. Discordances between phylogeny based on molecular markers and those based on morphological traits have been observed for various animal groups, including cnidarians [1]. Sea anemones (Cnidaria: Actiniaria) owe their phylogenetic uncertainty to the lack of correspondence between taxonomy and morphological and biological traits and the fact that phylogenetic relationships have not been rigorously explored through taxonomic classifications [2,3]. Although a variety of classifications have been proposed for relationships among Actiniaria [3,4,5,6], the phylogenetic relationships between several genera and species remain uncertain. While the column size, shape, and size of cnidae are usually useful for their taxonomy, such criteria are not always reliable taxonomic characteristics for proper phylogenetic classification of sea anemones [7]. Differences in cnidae have been considered a critical systematic value that accord with Actiniaria phylogeny [8]. Recent molecular studies have suggested that different clades vary in cnidae [9]. However, using differences in cnidae as a basis for classification can lead to incorrect phylogenetic placement of taxa, such as the revision of the acontiate actiniarians phylogeny that arose from molecular studies [1].

Arctic-boreal species of sea anemones from the family Actiniidae (Cnidaria, Anthozoa): *Aulactinia stella*, *Urticina crassicornis, Cribrinopsis similis*, that are found in aggregations in intertidal and subtidal zone of the Barents Sea show high plasticity for many morphological traits [10]. Sea anemones belonging to the genera *Actinia*, *Aulactinia*, *Anthopleura*, *Cribrinopsis,* and *Urticina* are sometimes wrongly identified. High similarity is observed in particular for *Urticina* and *Cribrinopsis*. Both genera are similar in their mostly decamerously arranged mesenteries and color patterns of specimens. Many species can coexist in the same habitat: *U. eques*, *U. crassicornis*, and *C. similis* in the subtidal zone of the Barents Sea, and *U. grebelyi*, *U. crassicornis*, and *C. albopunctata* in the north-western Pacific Ocean. Moreover, the taxonomic names of many genera have been changed or synonymized with others, which causes considerable confusion. This confusion has been observed for *Aulactinia,* which was previously named *Bunodactis* (Verrill, 1864) or *Cribrina* (McMurrich, 1910). Species of the genus *Urticina* have had extremely variable names and mistakes in their species identification. For instance, *Urticina crassicornis* (Müller, 1776) was previously named *Actinia crassicornis* (Müller, 1776), *Tealia crassicornis* (Carlgren, 1893), and *Urticina felina crassicornis* (Carlgren, 1921) and is still confused with *Urticina grebelnyi,* which is also called the Christmas or painted anemone [11]. Some species are not single but comprise several reproductively isolated sibling species, subspecies, or morphs, e.g., the common anemone along the coasts of Europe and the Mediterranean Sea—*Actinia equina* [12,13]. Alternatively, studies on the reproductive strategy of particular species resulted from misidentification. A well-known example is *Urticina crassicornis* (syn. *Tealia*) of Müller (1776), which was described as an internal brooder in contrast to *Urticina* (syn. *Tealia*) *crassicornis* of Gosse (1860), which releases its gametes freely and should most likely be identified as *Urticina felina* (Linnaeus, 1761).

In this study, we analyzed the phylogenetic relationships within the genera *Urticina*, *Cribrinopsis*, and *Aulactinia* based on mitochondrial 16S ribosomal DNA partial sequences, nuclear 28S ribosomal DNA partial sequences, and morphological and ecological data. We mostly collected individuals of internally brooding species from various sites of the Barents Sea and East Kamchatka and compared the usefulness of the mitochondrial and nuclear DNA sequences.

## 2. Materials and Methods

### 2.1. Sample Collection and Molecular Data Analysis

**Figure 1:**
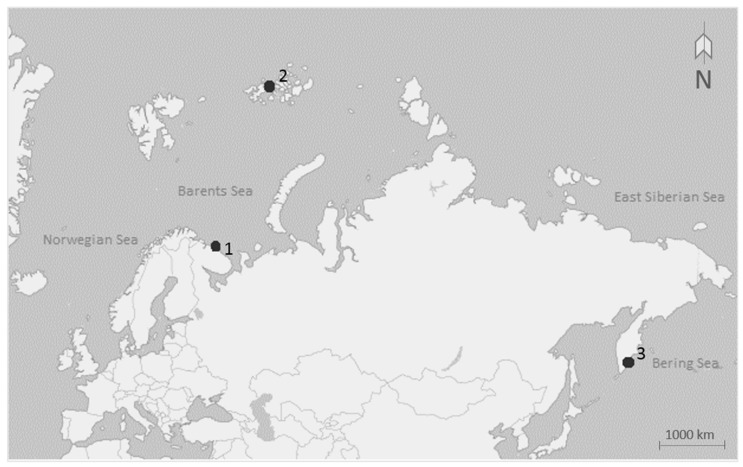
Map showing points of sample collection: (1) the coast of the Kola Peninsula 69° N, 36° W, (2) Franz Josef Land 80° N, 57° W, and (3) East Kamchatka, Starichkov Island 52° N, 158° E.

Sea anemones were collected from three different localities: (1) the coast of the Kola Peninsula 69° N, 36° W (intertidal and subtidal zone of the Barents Sea), (2) Franz Josef Land 80° N, 57° W (subtidal zone of the Barents Sea), and (3) East Kamchatka, Starichkov Island 52° N, 158° E (subtidal zone of the Pacific Ocean; Figure 1).

All specimens were identified using the distribution and size of cnidae in tentacles, actinopharynx, and columns. All the consensus sequences were submitted to the GenBank; their accession numbers are shown in Table 1 and Table 2, which summarize the species collected, sampling location, and variable color patterns of individuals within the *Urticina*, *Cribrinopsis*, and *Aulactinia* genera. The voucher specimens collected by A. Kaliszewicz, N. Panteleeva, I. Olejniczak, and P. Boniecki are stored at the Murmansk Marine Biological Institute, while others are deposited in private collections.

Small pieces of tissue (~20 mm^2^) were preserved in 96% ethanol for DNA analysis. DNA was isolated using a Genomic Mini AX Tissue Kit. DNA was amplified in a total volume of 25 μL of the following composition: 10 mM Tris-HCl, 50 mM KCl, 1.5 mM Mg^2+^, 0.001% gelatine, 200 μM dNTP, 2 U/μL Taq DNA Polymerase, 1 μL of primer (final concentration of 1 μM), 1.5 μL of MgCl2 (added to a final concentration of 3 mM), and 2 μL of DNA template (final concentration of 4 μg/mL). Ultra-purified water was used in the mixtures (TKA MicroPure UV). A pair of primers was used to amplify the partial 16S rDNA (5′-ACGGAATGAACTCAAATCATGT-3′, 5′-TCGACTGTTTACCAAAAACATA-3′; [14] and partial 28S rDNA (5′-ACCCGCTGAACTTAAGCA-3′, 5′-TCCTGAGGGAAACTTCGG-3′; [15]. The polymerase chain reaction (PCR) for the 16S rDNA region was conducted under the following conditions: 35 cycles, profile: 94 °C for 20 s, 50 °C for 45 s, and 68 °C for 120 s. The PCR for the 28S rDNA gene was performed using the following protocol: an initial denaturation at 94 °C for 3 min, 35 cycles of denaturation at 94 °C for 1 min, annealing at 40 °C for 20 s, elongation at 72 °C for 2 min, and a final elongation at 72 °C for 7 min. PCR products were purified using NucleoSpin Gel and PCR Clean-up (Macherey-Nagel). Partial 16S rDNA sequences were determined for 19 anthozoans. Partial 28S rDNA sequences were determined for 18 anthozoans. Sequences were assembled, edited, and annotated using the software programs Geneious ^®^11.1.2 [16] and BioEdit v7.2.5 Sequence Alignment Editor [17].

Members of three families were chosen as the outgroup: Metridinidae*—Metridium senile* (Linnaeus 1761), Actinostolidae*—Stomphia didemon* (Siebert 1973) and *Stomphia selaginella* (Stephenson 1918), and Hormathiidae*—Allantactis parasitica* (Danielssen 1890) and *Hormathia pectinata* (Hertwig 1882). Phylogenetic analyses were performed based on the sequences of partial 16S and 28S rDNA obtained from the collected specimens and sequences of additional Anthozoa obtained from GenBank. Sequences from GenBank were included as appropriate (Table 1 and Table 2).

Alignments were made using Clustal X [18]. The phylogenetic analyses were performed using Bayesian inference in MrBayes 3.2.6.

GTR+I+G was set as the evolutionary model. For each gene, a separate Bayesian analysis was run for 10 million generations, which were sampled at intervals of 1000 generations; the burn-in procedure involved 25% of the trees generated.

### 2.2. Ecological and Morphological Data Analysis

Ecological and morphological characters were selected for all species used in the molecular analyses. In total, 40 characters were analyzed (Appendix A) based on literature data [11,19,20,21,22,23,24,25,26]. The maximum parsimony (MP) analyses were conducted in TNT 1.5 [27] using the Traditional Search option to find the most parsimonious trees (MPTs) under the following parameters: memory set to hold 1,000,000 trees; tree bisection–reconnection (TBR) branch-swapping algorithm with 1000 replications saving 100 trees per replicate; zero-length branches collapsed after the search, with implied weighting option with the concavity (K) value set to 3. Character mapping was conducted in Winclada v1.00.08 [28] using unambiguous optimization. All characters were treated as unordered and analyses were performed under equal weights. The analysis was set to find the minimum tree length. An outgroup served as the same species that were used in the molecular analysis (Table 1, Table 2 and Table S1).

## 3. Results

### 3.1. Alignments and Trees Based on Molecular Characteristics

**Figure 2:**
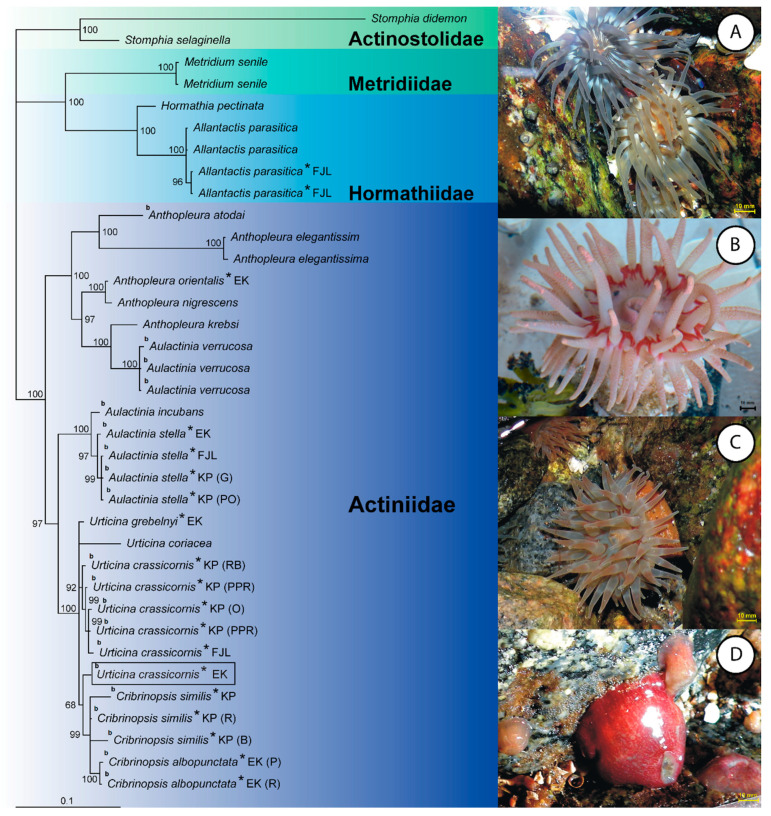
Neighbor-joining tree from 28S rDNA sequences. The asterisk indicates the sample analyzed in this study, the others are taken from GenBank. Letters next to the species name indicate the collection site of species reported in this study (KP—Kola Peninsula, FJL—Franz Josef Land, EK—East Kamchatka). The color pattern for specimens of *Urticina crassicornis, Cribrinopsis similis, Cribrinopsis albopunctata*, and *Aulactinia stella* are given in parenthesis (PPR—pale pink with red stripes, PP—pale pink, RB—red–blue blotched, PO—pale orange, OG—orange with gray tentacles, O—orange, R—red, G—gray, B—beige). The color pictures: A—two morphs (gray—G and pale orange—PO) of *Aulactinia stella,* B—*Cribrinopsis similis* (beige—B), C—*Urticina crassicornis* (orange—O), D—*Urticina crassicornis* (red—R). Numbers at nodes are posterior probability values. The letter b indicates the species with the brooding strategy. The framed name *Urticina crassicornis* from the Pacific is probably a different species.

This study obtained 19 partial 28S rDNA fragments with lengths ranging from 854 to 930 base pairs (bp). The final alignment of 36 sequences resulted in a total length of 1005 bp. In the consensus Bayesian tree based on this nuclear marker, the genus *Aulactinia* does not form a single clade but instead is a sister group to the *Urticina* and *Cribrinosis* groups (Figure 2).

The exception is *Aulactinia verrucosa,* which is paired with *Anthopleura krebsi*. This relationship is well supported by a posterior probability (PP) of 100%. In the mitochondrial tree, *A. verrucosa* is also found among *Anthopleura* species not within the *Aulactinia* clade. The nuclear dataset revealed that the systematic position of another species, *Urticina crassicornis* from East Kamchatka, also needs revision. This Pacific species was not positioned within the *Urticina* group (which includes *U. crassicornis* from Europe, *U. grebelnyi* from East Kamchatka, and *U. coriacea*) but as a member of the sibling *Cribrinopsis* group (Figure 2). Another Pacific species of the Actiniidae family, *Aulactinia stella* from East Kamchatka, was a member of the *Aulactinia* group, a classification in line with current taxonomy (Figure 2).

**Figure 3:**
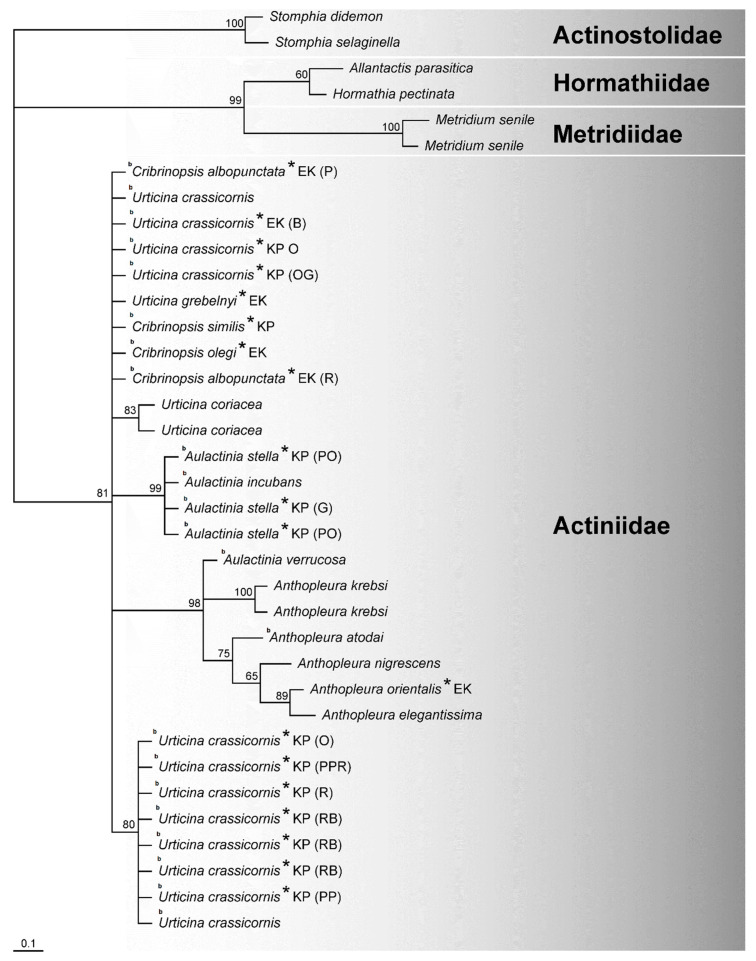
Neighbor-joining tree from 16S rDNA sequences. The sequences with the numbers were taken from GenBank. Letters next to the species name indicate the collection site of species reported in this study (KP—Kola Peninsula, EK—East Kamchatka). The color patterns for specimens of *Urticina crassicornis, Cribrinopsis similis, Cribrinopsis albopunctata,* and *Aulactinia stella* are given in parentheses (PPR—pale pink with red stripes, PP—pale pink, RB—red–blue blotched, PO—pale orange, OG—orange with gray tentacles, O—orange, R—red, G—gray, B—beige). Numbers at nodes are posterior probability values. The letter b indicates the species with the brooding strategy.

The specimens belonging to *A. stella*, an internal brooding species of the Actiniidae family collected from the Barents Sea near Franz Josef Land and the Kola Peninsula, were closely related; their position was supported by a PP of 99% (Figure 2). Similar results (PP value of 99%) were obtained for specimens of *U. crassicornis*, another species described as an internal brooder, collected from these two sites. Specimens with identical color patterns (Table 1 and Table 2) were not positioned together in both the nuclear and mitochondrial trees (Figure 2 and Figure 3).

The 16S and 28S rDNA datasets were analyzed separately. The topology of the 16S rDNA tree is not congruent with that of the 28S rDNA tree (Figure 2 and Figure 3). The 19 partial 16S rDNA fragments obtained in this study ranged from 447 to 504 bp in length; the total length of the final alignment was 572 bp. The alignment of all 36 sequences revealed that the 16S rDNA region is very conservative. The general topology of the tree shows that taxa belonging to the four analyzed family groups were relatively well separated, with PP values ranging from 81 to 100%. Within Actiniidae, well-supported clades were formed by specimens of *Aulactinia stella* and *A. incubans* (PP 99%) and for *Anthopleura* species together with *Aulactinia verrucosa* (PP 98%). The remaining taxa have unresolved positions. The 16S rDNA appeared insufficient for phylogenetic analyses at the lower taxonomic levels of Actiniaria. In contrast, the genera based on the nuclear sequences were well separated on the tree. The 28S dataset did not represent *Anthopleura* as a monophyletic group with a singlet taxon: *Anthopleura atodai* and *A. elegantissima* (Figure 2). The species belonging to the families Actinostolidae, Hormathiidae, and Metridiidae formed distinct groups from Actiniidae in the nuclear and mitochondrial trees (Figure 2 and Figure 3). These results suggest that the nuclear 28S rDNA is more variable and appropriate for phylogenetic studies of the Actiniidae family than mitochondrial 16S rDNA (Figure 2 and Figure 3).

### 3.2. Trees Based on Morphological and Ecological Characters

**Figure 4:**
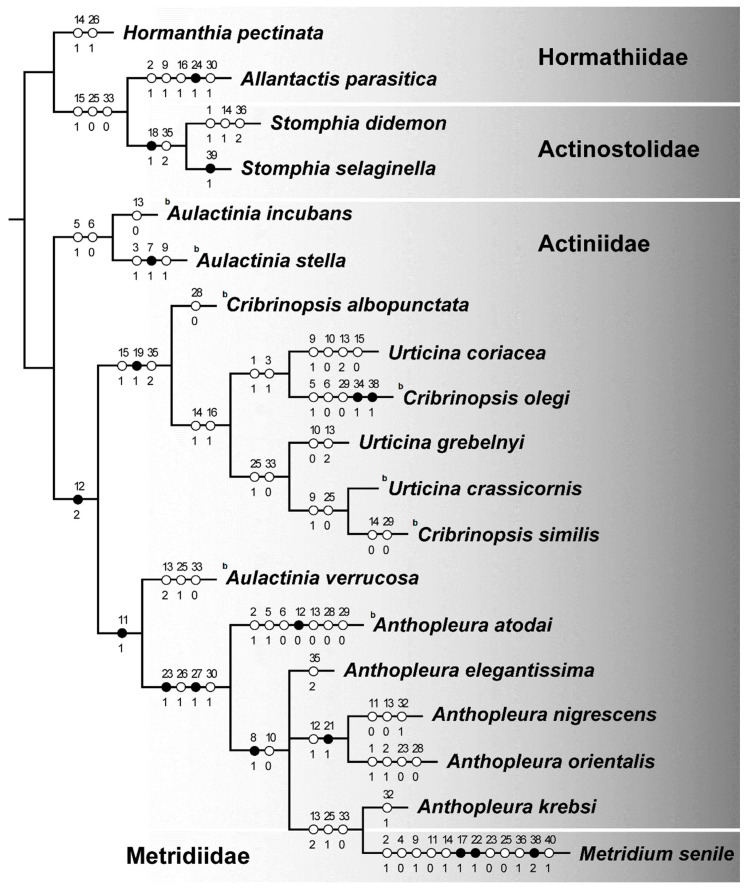
Maximum parsimony tree based on morphological and ecological data. The letter b indicates the species with the brooding strategy.

We obtained a single most parsimonious tree which did not coincide with the trees based on molecular data. The species belonging to the family Hormathiidae did not form a distinctive group (Figure 4).

Contrary to the nuclear tree, the genera *Aulactinia* and *Anthopleura* are members of a sister group to genera *Urticina* and *Cribrinopsis*, which were also positioned together in the molecular trees.

On the other hand, we found confirmation of the results of the molecular trees. *Aulactinia verrucosa* is found among *Anthopleura* species not within the *Aulactinia* clade. Both the mitochondrial tree and the tree based on nuclear 28S rDNA regions indicated a close relationship between *Anthopleura* species and *Aulactinia verrucosa*, which is not a sister to other *Aulactinia* (*A. incubans* and *A. stella*). The ecological and morphological datasets do not show monophyly of the genus *Anthopleura* concordant to the molecular results based on the nuclear sequences.

## 4. Discussion

The phylogeny based on nuclear 28S rDNA sequences allowed for the proper classification of *Aulactinia* and *Anthopleura*. However, 28S rDNA did not confirm the validity of the separate genera *Urticina* and *Cribrinopsis*. Species belonging to *Urticina* are similar in morphological traits, color patterns, and habitat preference to specimens of the genus *Cribrinopsis*. They exhibit similar reproductive strategies. Species belonging to *Urticina* (*U. crassicornis*) as well as to *Cribrinopsis* (*C. albopunctata*, C*. fernaldi*, *C. olegi*, *C. similis*) are known as internal brooders. For this reason, the results based on the analyses of morphological and ecological characteristics of genera (*Urticina*–*Cribrinopsis*) placed them within one group and appeared insufficient for their separation into sister taxa.

The tree based on 28S rDNA sequences revealed inconsistency between specimens determined to be European *Urticina crassicornis* and Pacific *U. crassicornis*. The latter seems to be more closely related to *Cribrinopsis* than *Urticina*; more molecular analyses and possible taxonomic revision are needed. In contrast to Pacific *U. crassicornis*, the specimens collected from different localities in the Barents Sea (Kola Peninsula and Franz Josef Land, about 1300 km distant from each other in a straight line) are on the same tree branch. Similarly, specimens of *Aulactinia stella* from these two localities are paired together. Both species brood their young internally. This reproductive mode has been described as increasing offspring survival but decreasing dispersal potential [29,30]. The dispersal of sea anemones with brooding juveniles seems to be less limited than expected. Similar genetic structures have been demonstrated in the aggregations of *A. stella* and *U. crassicornis* inhabiting the White and Barents Seas. The haplotypes detected among specimens of *A. stella* from sites about 700 km away from each other were the same, contrary to haplotypes recovered in the population from the Pacific Ocean [31]. These results suggest that the dispersion of the juveniles of the brooding species might be sufficient to settle habitats more than a thousand kilometers away.

The topology of the nuclear and morphological trees evidenced the polyphyly of the genus *Anthopleura* and confirmed existing reports that *Anthopleura* is not a monophyletic genus [2,24,32]. Previous studies based on mitochondrial (12S, 16S rDNA, COIII) and nuclear (ITS, 28S rDNA) markers revealed the broad polyphyly of *Anthopleura*, a group with species of *Bunodosoma*, *Aulactinia*, *Anthostola,* and *Bunodactis* [2,32]. In this study, the trees based on nuclear 28S rDNA regions indicated a close relationship between *Anthopleura krebsi* and *Aulactinia verrucosa*, which is not a sister to other *Aulactinia* (*A. incubans* and *A. stella*). The tree based on morphological and ecological characters also indicated *A. verrucosa* among *Anthopleura* species not within the *Aulactinia* clade. These results suggest either polyphyly of the *Aulactinia* genus or incorrect naming and classification of the *A. verrucosa* synonym *Bunodactis verrucosa* (Pennant, 1777). Such findings are consistent with the proposition of Daly et al. [32] that all species belonging to *Bunodactis* cannot be renamed as *Aulactinia*. Specimen variations in coloration, column size, shape, and size of cnidae can lead to misidentification or incorrect taxonomic classification, as indicated in the nuclear tree. Morphological traits do not seem to be a reliable indicator of phylogenetic divergence of species or even genera. Moreover, the results revealed that 16S rDNA sequences are also insufficient for separating taxa lower than the level of families. Low usefulness of 16S rDNA fragment for studies of actiniarian diversity was indicated by other studies [33,34]. The molecular methods used do not allow for the identification of individuals with a specific color. Our results reveal that further studies based on nuclear sequences are needed to confirm the separation of genera, such as *Urticina* and *Cribrinopsis*.

The comparison of genealogies based on different datasets showed the sequencing of nuclear genes as desirable for analyses of taxonomic relationships between genera *Aulactinia*, *Urticina,* and *Cribrinopsis*. Mitochondrial and morphological genealogies did not appear to be representative and sufficient to analyze sea anemones’ evolutionary history, a finding that supports already existing findings for Anthozoa [35]. Contrary to other animals, mitochondrial DNA of primitive metazoans, such as sponges and anthozoans, has been described as slowly evolving, invariant among conspecifics, and of limited use for taxonomic studies [36,37]. There are only a few regions of anthozoan mitochondrial DNA that exhibit enough variability to separate congeneric species (e.g., the putative control region), while commonly used *COI*, 16S rDNA, and cytochrome b sequences exhibit low levels of divergence [36].

## 5. Conclusions

The sequencing of nuclear genes allowed for analysis of taxonomic relationships between genera of the Actiniidae family, e.g., *Aulactinia*, *Urticina,* and *Cribrinopsis*, containing internally brooding species inhabiting the Arctic-boreal region. These sea anemones provide many taxonomic problems and still require phylogenetic revision. Morphological traits appeared to be not reliable taxonomic characteristics for the separation of closely related sea anemone species. Our tree based on 16S rDNA mitochondrial sequences confirmed the conclusions from the literature—insufficient for separating taxa lower than the level of families. Despite the widespread opinion on the relationship between low dispersion and brooding strategy, the dispersion of the juveniles of the studied Arctic-boreal species might be sufficient to settle habitats more than a thousand kilometers away.

## Figures and Tables

**Table 1:** Species analyzed phylogenetically based on partial sequences of the mitochondrial 16S rDNA gene. The collection site of species reported in this study (KP—Kola Peninsula, EK—East Kamchatka) and color patterns for specimens of species variable in their coloration: *Urticina crassicornis*, *Cribrinopsis similis*, *C. albopunctata*, and *Aulactinia stella*, are as follows: (PPR—pale pink with red stripes, PP—pale pink, RB—red–blue blotched, PO—pale orange, OG—orange with gray tentacles, O—orange, R—red, G—gray, B—beige). * sequences reported in this study (Clustal X), - no data.

Class	Family	Species	Locality	Color Pattern	GenBank Accession
					Mitochondrial 16S rDNA
Anthozoa	Actiniidae	*Urticina grebelnyi*	EK		MW491942 *
		*Urticina coriacea*			EU190797
					KT852114
		*Urticina crassicornis*	KP	RB	MW491943 *
			KP	OG	MW491944 *
			KP	PPR	MW491945 *
			KP	O	MW491946 *
			KP	RB	MW491947 *
			KP	PP	MW491948 *
			KP	O	MW491949 *
			EK	B	MW491950 *
			KP	RB	MW491951 *
			KP	R	MW491952 *
					KT85205
					JQ92744
		*Cribrinopsis albopunctata*	EK	PP	MW491953 *
			EK	R	MW491954 *
		*Cribrinopsis olegi*	EK		MW491955 *
		*Cribrinopsis similis*	KP	-	MW491956*
		*Aulactinia incubans*			KT852080
		*Aulactinia stella*	KP	G	MW491959 *
			EK	-	MW491958 *
			KP	PO	MW491957 *
		*Aulactinia verrucosa*			EU190766
		*Anthopleura atodai*			KT852055
		*Anthopleura elegantissima*			AEU40292
		*Anthopleura krebsi*			EU190758
					KY789339
		*Anthopleura nigrescens*			KY789340
		*Anthopleura orientalis*	EK		MW491960 *
	Actinostolidae	*Stomphia didemon*			EU190795
		*Stomphia selaginella*			GU473298
	Hormathiidae	*Allantactis parasitica*			FJ489420
		*Hormathia pectinata*			FJ489430
	Metridiidae	*Metridium senile*			AY345876
					EU190786

**Table 2:** Species analyzed phylogenetically based on the partial sequences of nuclear 28S rDNA gene. The collection site of species reported in this study (KP—Kola Peninsula, FJL—Franz Josef Land, EK—East Kamchatka) and color pattern for specimens of species that varied in their coloration: *Urticina crassicornis*, *Cribrinopsis similis*, *C. albopunctata*, and *Aulactinia stella*, are given (PPR—pale pink with red stripes, PP—pale pink, RB—red–blue blotched, PO—pale orange, OG—orange with gray tentacles, O—orange, R—red, G—gray, B—beige). * sequences reported in this study (Clustal X), - no data.

Class	Family	Species	Locality	Color Pattern	GenBank Accession
					Nuclear 28S rDNA
Anthozoa	Actiniidae	*Urticina grebelnyi*	EK		MW491984 *
		*Urticina coriacea*			KT852266
		*Urticina crassicornis*	KP	PPR	MW491985 *
			KP	RB	MW491986 *
			KP	O	MW491987 *
			KP	PPR	MW491988 *
			FJL	-	MW491989 *
			EK	-	MW491990 *
		*Cribrinopsis albopunctata*	EK	R	MW491991 *
			EK	PP	MW491992 *
		*Cribrinopsis similis*	KP	R	MW491993 *
			KP	B	MW491994 *
			KP	-	MW491995 *
		*Aulactinia incubans*			KT852256
		*Aulactinia stella*	EK	-	MW491996 *
			KP	G	MW491997 *
			KP	PO	MW491998 *
			FJL	-	MW491999 *
		*Aulactinia verrucosa*			EU190812
					KJ483084
					KT852250
		*Anthopleura atodai*			KT852247
		*Anthopleura elegantissima*			EU190801
					KJ483104
		*Anthopleura krebsi*			EU190804
		*Anthopleura nigrescens*			KY789375
		*Anthopleura orientalis*	EK		MW492000 *
	Actinostolidae	*Stomphia didemon*			EU190837
		*Stomphia selaginella*			GU473331
	Hormathiidae	*Allantactis parasitica*	FJL		MW492001 *
					MW492002 *
			FJL		FJ489454
					KJ483056
		*Hormathia pectinata*			FJ489465
	Metridiidae	*Metridium senile*			EU190829
					JF833000

## Data Availability

Not applicable.

## Supplementary Materials

The following are available online at https://www.mdpi.com/2079-7737/10/2/81/s1, Table S1: Ecological and morphological data matrix with binary state characteristics used in building of the morphological tree.

## Abbreviations

16S rDNA	the sequence of mitochondrial small subunit of a ribosome
28S rDNA	the sequence of nuclear subunit of a ribosome
B	color of sea anemones, beige
CP	Central Poland
EK	East Kamchatka
FJL	Franz Josef Land
G	color of sea anemones, grey
KP	Kola Peninsula
O	color of sea anemones, orange
OG	color of sea anemones, orange with grey tentacles
PCR	polymerase chain reaction
PO	color of sea anemones, pale orange
PP	color of sea anemones, pale pink
PPR	color of sea anemones, pale pink with red stripes
R	color of sea anemones, red
RB	color of sea anemones, red–blue blotched.

## Appendix A. Ecological and Morphological Characters Used in the Phylogenetic Analysis

1. Habitat preference: rocks (0), sand (1).
2. Occurrence on shells: absent (0), present (1).
3. Buried in sand: no (0), yes (1).
4. Intertidal zone: not inhabited (0), inhabited (1).
5. Hermaphroditism: not observed (0), observed (1).
6. Dioecy: not observed (0), observed (1).
7. Parthenogenesis: not observed (0), observed (1).
8. Asexual reproduction (budding, fission, pedal laceration): not observed (0), observed (1).
9. In Arctic waters: absent (0), present (1)
10. Brooding: not observed (0), observed (1).
11. Symbionts: absent (0), present (1).
12. Column diameter: small size <1.0 cm (0), medium size 1.0–5.0 cm (1), large size >5.0 cm (2).
13. Column high: <3.0 cm (0), 3.0–8.0 cm (1), >8.0 cm (2).
14. Number of tentacles: ≤ 96 (0), >96 (1).
15. Spirocysts in tentacles: <50 µm (0), ≥ 50 µm (1).
16. Basitrichs in tentacles: <30 µm (0), ≥ 30 µm (1).
17. p-mastigophores in tentacles: absent (0), present (1).
18. b-mastigophores in tentacles: absent (0), present (1).
19. Basitrichs in actinopharynx: <60 µm (0), ≥ 60 µm (1).
20. p-mastigophores in actinopharynx: absent (0), present (1).
21. p-mastigophores in actinopharynx: <30 µm (0), ≥ 30 µm (1).
22. Differences in cnidae between feeding-tentacles and catch-tentacles: not observed (0), observed (1).
23. Dense aggregations: not formed (0), formed (1).
24. Mutually relationships with gastropods: absent (0), present (1).
25. Verrucae: absent (0), nonadhesive (1), adhesive (2).
26. Acrorhagi: absent (0), present (1).
27. Aggressive behavior: not observed (0), observed (1).
28. Color of oral disc: similar to the column (0), different from the column (1).
29. The pedal disc: similar in diameter to the column (0), wider than the column (1).
30. The tentacles are: the same color as the disc (0), different in color from the disc (1).
31. Inhabitance of the littoral zone: absent (0), present (1).
32. Tropical seas: not occupied (0), occupied (1).
33. Body wall: without adhered particles (0), with adhered particles (sand, gravel, shell) (1).
34. Mutual relationships with shrimps: absent (0), present (1).
35. Color of the column: pale (0), colorful without stripes or spots (1), colorful with stripes or spots (2).
36. Color of the tentacles: pale (0), colorful (1), striped or spotted (2).
37. Arrangement of tentacles: scattered (0), one cycle (1), more than two cycles (2).
38. Shape of tentacles: conical (0), spherical (1), slender (2).
39. In Antarctic waters: absent (0), present (1)
40. Color of the oral disc: pale (0), colorful without stripes or spots (1), colorful with stripes or spots (2)
