# Supplementary material for: Phylogenetic Relatedness within the Internally Brooding Sea Anemones from the Arctic-Boreal Region
Source: Biology (Basel). 2021 Jan 22;10(2):81. doi: 10.3390/biology10020081 (PMC7911183; doi:10.3390/biology10020081)
Supplement: Supplementary file 1 [file biology-10-00081-s001.pdf]

biology

MDPI

Supplementary Material

Phylogenetic Relatedness within the Internally Brooding Sea Anemones from the Arctic-Boreal Region

Anita Kaliszewicz, Ninel Panteleeva, Magdalena Żmuda-Baranowska, Karol Szawaryn, Izabella Olejniczak, Paweł Boniecki, Sergey D. Grebelnyi, Dagmara Kabzińska, Jerzy Romanowski, Rafał Maciaszek, Ewa B. Górska and Joanna Zawadzka-Sieradzka

Table S1. Ecological and morphological data matrix with binary state characteristics used in building of the morphological tree.

Species	Ecological and Morphological Characters																																							
	1	2	3	4	5	6	7	8	9	10	11	12	13	14	15	16	17	18	19	20	21	22	23	24	25	26	27	28	29	30	31	32	33	34	35	36	37	38	39	40
Urticina grebelnyi	0	0	0	0	0	1	0	0	0	0	0	2	2	1	1	1	0	0	1	1	0	0	0	0	1	0	0	1	1	0	1	0	0	0	2	2	2	0	0	2
Urticina coriacea	1	0	1	1	0	1	0	0	1	0	0	2	2	1	0	1	0	0	1	1	0	0	0	0	2	0	0	1	1	0	1	0	1	0	2	2	2	0	0	2
Urticina crassicornis	0	0	0	1	0	1	0	0	1	1	0	2	1	1	1	1	0	0	1	1	0	0	0	0	0	0	0	1	1	0	1	0	0	0	2	2	2	0	0	2
Cribrinopsis albopunctata	0	0	0	0	0	1	0	0	0	1	0	2	1	0	1	0	0	0	1	1	0	0	0	0	2	0	0	0	1	0	1	0	1	0	2	2	2	0	0	2
Cribrinopsis olegi	1	0	1	0	1	0	0	0	0	1	0	2	1	1	1	1	0	0	1	1	0	0	0	0	2	0	0	1	0	0	1	0	1	1	2	2	2	1	0	2
Cribrinopsis similis	0	0	0	1	0	1	0	0	1	1	0	2	1	0	1	1	0	0	1	1	0	0	0	0	0	0	0	1	0	0	1	0	0	0	2	2	2	0	0	2
Aulactinia incubans	0	0	0	1	1	0	0	0	0	1	0	1	0	0	0	0	0	0	0	0	0	0	0	0	2	0	0	1	1	0	1	0	1	0	1	1	2	0	0	2
Aulactinia stella	0	0	1	1	1	0	1	0	1	1	0	1	1	0	0	0	0	0	0	1	0	0	0	0	2	0	0	1	1	0	1	0	1	0	1	2	2	0	0	2
Aulactinia verrucosa	0	0	0	1	0	1	0	0	0	1	1	2	2	0	0	0	0	0	0	0	0	0	0	0	1	0	0	1	1	0	1	0	0	0	1	2	2	0	0	2
Anthopleura atodai	0	1	0	1	1	0	0	0	0	1	1	0	0	0	0	0	0	0	0	0	0	0	1	0	2	1	1	0	0	1	1	0	1	0	1	2	2	0	0	2
Anthopleura elegantissima	0	0	0	1	0	1	0	1	0	0	1	2	1	0	0	0	0	0	0	0	0	0	1	0	2	1	1	1	1	1	1	0	1	0	2	2	2	0	0	2
Anthopleura krebsi	0	0	0	1	0	1	0	1	0	0	1	2	2	0	0	0	0	0	0	0	0	0	1	0	1	1	1	1	1	1	1	1	0	0	1	2	2	0	0	2
Anthopleura nigrescens	0	0	0	1	0	1	0	1	0	0	0	1	0	0	0	0	0	0	0	0	1	0	1	0	2	1	1	1	1	1	1	1	1	0	1	2	2	0	0	2
Anthopleura orientalis	1	1	0	1	0	1	0	1	0	0	1	1	1	0	0	0	0	0	0	0	1	0	0	0	2	1	1	0	1	1	1	0	1	0	1	2	2	0	0	2
Stomphia didemon	1	0	0	0	0	1	0	0	0	0	0	1	1	1	1	0	0	1	0	1	0	0	0	0	0	0	0	0	1	0	0	0	0	0	2	2	2	0	0	1
Stomphia selaginella	0	0	0	0	0	1	0	0	0	0	0	1	1	0	1	0	0	1	0	0	0	0	0	0	0	0	0	0	1	0	0	0	0	0	2	1	2	0	1	1
Allantactis parasitica	-	1	0	0	0	1	0	0	1	0	0	1	1	0	1	1	0	0	0	1	0	0	0	1	0	0	0	0	1	1	0	0	0	0	1	1	2	0	0	1
Hormathia pectinata	0	0	0	0	0	1	0	0	0	0	0	1	1	1	0	0	0	0	0	0	0	0	0	0	2	1	0	0	1	0	0	0	1	0	1	1	2	0	0	1
Metridium senile	0	1	0	0	0	1	0	1	1	0	0	2	2	1	0	0	1	0	0	0	0	1	0	0	0	1	1	1	1	1	1	0	0	0	1	1	2	2	0	1
